# Agronomic Traits, Fresh Food Processing Characteristics and Sensory Quality of 26 Mung Bean (*Vigna radiata* L.) Cultivars (Fabaceae) in China

**DOI:** 10.3390/foods11121687

**Published:** 2022-06-08

**Authors:** Tianyao Zhao, Xiao Meng, Chen Chen, Lixia Wang, Xuzhen Cheng, Wentong Xue

**Affiliations:** 1 College of Food Science and Nutritional Engineering, China Agricultural University, Beijing 100083, China; zty@cau.edu.cn (T.Z.); mxiao@cau.edu.cn (X.M.); 15515658204@163.com (C.C.); 2 Institute of Crop Sciences, Chinese Academy of Agricultural Sciences, Beijing 100081, China; wanglixia03@caas.cn

**Keywords:** mung bean, yield, fresh food processing, sensory quality, grey relational analysis

## Abstract

In recent years, with the expansion of mung bean (*Vigna radiata* L.) planting areas and the increase of consumer demand, it has become imperative to screen high-quality mung bean cultivars. In this study, the agronomic traits, fresh bean characteristics, and sensory evaluation of boiled beans were analyzed for 26 mung bean cultivars. The results showed that the variation coefficient and genetic diversity index of six agronomic traits of mung bean ranged from 9.04% to 44.98%, 1.68 to 1.96, respectively, with abundant genetic variation, and the highest was the grain yield. Mung bean cultivars with higher grain yield had more advantage in the number of branches, number of pods per plant, and 100-seed weight. The fresh bean traits were relatively stable, with an average coefficient variation of 8.48%. The trait with the highest genetic diversity index was the number of seeds per pod (2.03). The cultivar with the highest total sensory evaluation score of boiled beans was Zhanglv 3 (75.67), which had more advantages in taste and color. Through the comprehensive evaluation of grey relational analysis, the cultivars suitable for fresh food processing were Zhonglv 3 (0.960), Jilv 11 (0.942), Zhonglv 1 (0.915), CES-78 (0.899) and Kelv 2 (0.896). Generally, the high-quality cultivars with higher yield and fresh food processing characteristics were CES-78, Kelv 2, Zhonglv 16, and Zhonglv 2. This study provided a preference for the breeding of fresh mung bean cultivars, development of new products and improvement of mung bean resource utilization.

## 1. Introduction

Mung bean (*Vigna radiata* L.), alternatively known as green gram or moong bean, belongs to the *Fabaceae* botanical family and the genus *Vigna* with chromosome number 2n = 22 [1,2]. As an important economic legume crop, mung bean possesses wide adaptability, strong stress resistance, short growth period, long sowing period, etc. It often improves soil fertility and productivity through nodule nitrogen fixation and fertilization [3,4]. In China, mung bean is a major variety of food legumes and mainly planted in Northeast China and Huang-Huai-Hai Plain [5], and has been cultivated for more than 2000 years [6]. The total yield and export volume of mung beans in China ranks first in the world, with a total annual harvest of 1 million tons and exports of about 150,000~250,000 tons [7].

With the improvement of people’s living standards and the diversification of dietary structure, fresh beans are generally welcomed because of their rich nutritional value, and their consumption demand is increasing year by year. Fresh mung beans are rich in nutritional value [8,9]; they are a high-quality natural source of plant protein (the average content is 21.6 g/100 g), used as a substitute for meat and milk protein in many underdeveloped countries [10,11]. In addition, mung bean also contains a variety of essential amino acids (methionine, tryptophan, tyrosine, etc.), carbohydrates (55.6 g/100 g), dietary fiber (6.4 g/100 g), minerals (iron, phosphorus, sodium, calcium, potassium, magnesium, etc.), and a large number of biologically active substances (flavonoids, alkaloids, tannins) [12,13,14], which have physiological functions such as antioxidation, antitumor, antibiosis, anti-obesity, reducing blood fat, detoxification activities, relieving heatstroke and regulating gastrointestinal discomfort [15,16]. Boiled mung beans have a good taste, and are often promoted as functional foods, beneficial to human health [17].

With the continuous expansion of mung bean planting areas and the adjustment of agricultural structure, the demand for mung bean in domestic and foreign markets has gradually increased, which makes it imperative to screen mung bean cultivars with high-quality, high yield and processing adaptability. In order to meet the market demand, it is necessary to study the genetic diversity of mung bean germplasm resources. Based on the identification of its agronomic traits (yield, plant height, number of branches, 100-seed weight, etc.), it is important to evaluate its fresh food processing characteristics, which is not only conducive to the breeding of modern mung bean cultivars and promoting the research and development of mung bean products, but also can enhance the international competitiveness of mung bean and its products and promote the sustainable development of the mung bean industry.

Up to now, the National Crop Gene Bank of China has documented more than 5000 mung bean accessions [5]. Although there are abundant mung bean germplasm resources in China, the current research mainly focuses on mung bean breeding, cultivation techniques, and pest control [18,19,20]. In contrast, the research on the selection of mung bean cultivars with fresh food processing characteristics is relatively scarce. In this study, 26 mung bean cultivars were selected, and their agronomic traits were identified and analyzed during the fresh picking and harvest periods; sensory evaluation of boiled beans was studied, aiming at screening mung bean cultivars with high yield and excellent fresh food processing characteristics, and providing a scientific basis for the evaluation and application of mung bean cultivars.

## 2. Materials and Methods

### 2.1. Plant Materials and Experiment Design

Twenty-six mung bean cultivars were collected from the China National Crop Germplasm Genebank (Table 1). The seeds had been removed from impurities before sowing. All cultivars were planted in the experimental plot of the Institute of Crop Sciences, Chinese Academy of Agricultural Sciences (40°13′ N, 116°33′ E) and sown on 24 June 2020. The average annual temperature, precipitation and sunshine duration was 11.5 °C, 625 mm, and 2759 h, respectively. Each cultivar was sown in 2 rows, and the row length, row spacing and plant spacing were 2 m, 0.5 m and 0.15 m, respectively. The water and fertilizer management conditions were consistent throughout the growth period. The emergence, florescence and maturity of cultivars are shown in Table S1.

### 2.2. Sample Collection and Trait Investigation

The fresh mung bean pods were picked in batches during the tender stage and the pods which were not full, disease spots and insect spots were removed. The picking standard is that the pods are a bright green color, the grains are plump and swollen, and they are not overripe. For the maturity date of mung bean, its growth period, plant height, number of branches, number of pods per plant, grain yield per plant, and 100-seed weight were measured. The investigation standard of mung bean field agronomic traits refers to “Descriptors and data standard for mung bean (*Vigna radiata* L.)” [21].

### 2.3. Determination of Pod Weight, Length, Diameter and the Number of Seeds per Pod

Using an analytical balance to weigh the fresh pod, results were expressed as g of sample. The UA Vernier caliper was used to measure the length and width of fresh pods, using mm as the unit of measurement. After the fresh pods were peeled off, the fresh beans were taken out for determination, and the number of seeds per pod was obtained by counting method. Results were expressed as percentage of sample.

### 2.4. Determination of the Fresh and Boiled Bean Hardness

The picked fresh pods were put into food-grade non-woven bags and were boiled in 1000 mL boiling water for 5 min. The hardness of fresh and boiled beans was measured by a CT3 physical property analyzer, the test condition was 50% deformation, and the test speed was 1 mm/s. The TA39 probe fixture was not used [17,22]. Results were expressed as Newtons (N) of sample.

### 2.5. Sensory Evaluation of Boiled Beans

Sensory evaluation is one of the most intuitive methods to capture consumers’ perception of product quality, which involves measuring, evaluating, and construing the sensory responses for the taste, smell or touch of food [23]. Ten people with professional knowledge and experience in sensory evaluation were invited to grade the sensory quality of boiled beans according to sensory standards. Sensory evaluation of boiled mung bean was carried out in terms of aroma, color and luster, organizational form and taste [24,25]. The specific scoring criteria are shown in Table S2.

### 2.6. Establishment of Grey Relational Degree Model

In this study, mung bean cultivars represent a grey system, with each mung bean variety considered as a factor in the system. Pod weight, pod length, pod diameter, number of seeds per pod, fresh bean hardness, aroma, color and luster, organizational form and taste were correlated with fresh food characteristics of mung bean cultivars, which was regarded as a positive correlation index, while boiled bean hardness was a negative correlation index. The reference sample is a calculated ideal variety, from the best merchandise, its positive correlation index 5% higher than the maximum data value of the tested sample, and its negative correlation index 5% lower than the minimum value [26]. By calculating the weighted correlation degree between ideal cultivars and tested cultivars, different mung beans were compared. A high correlation coefficient is indicative that the degree of similarity between the sample and the ideal sample is high. The correlation coefficient was calculated according to the method reported by Kadier [27]. The data processing method was as follows: first, the ratio of original data to reference data was calculated to obtain dimensionless data to ensure the equivalence of all data. Secondly, assuming that the ideal list was X0, the compared list was Xi, i = 1, 2, 3 …, and X0 = {X0(1), X0(2), X0(3) … X0(k)}, Xi = {Xi(1), Xi(2), Xi(3) … Xi(k)}, k = 1, 2, 3…M. The correlation coefficient between the samples and the ideal sample at the *k* point was calculated using the following equation:

(1) $$\zeta i\left(k\right)=\frac{\min\min\left|\Delta i\left(k\right)\right|+\rho \max\max\left|\Delta i\left(k\right)\right|}{\left|\Delta i\left(k\right)\right|+\rho \max\max\left|\Delta i\left(k\right)\right|}$$

While Δi(k) = |X0(k)-Xi(k)|, min|Δi(k)| is the minimum value based on different k values, min min|Δi(k)| is the minimum value based on different i values, the same as max|Δi(k)| and max max|Δi(k)|. In Equation (1), the coefficient ρ (0 ≤ ρ ≤ 1) was used to increase the significance of the difference, which was set to 0.5, because this value offers moderate distinguishing effects and good stability. The average grey correlation coefficient at the k point was determined by Equation (2):

(2) $$\gamma k=\frac{1}{N}\overset{n}{\underset{i=1}{\sum }}\zeta i\left(k\right)$$

The weighting coefficient k was calculated by Equation (3):

(3) $$\mathrm{Wk}=\frac{\gamma k}{\sum_{1}^{M}\gamma k}$$

The grey relational degree of factor i was determined by Equation (4):

(4) $$\mathrm{Gi}=\overset{M}{\underset{k=1}{\sum }}\zeta i\left(k\right)\mathrm{Wk}$$

### 2.7. Statistical Analysis

The values of quantitative traits were standardized and classified according to 10 grades, grade 1 < X − 2δ, grade 10 ≥ X + 2δ, and the difference between each grade in the middle was 0.5δ, δ being the standard deviation. The phenotypic traits of mung bean were statistically analyzed by Excel, and the mean, standard deviation and coefficient of variation of each trait were calculated. The genetic diversity of mung bean cultivars was calculated by the Shannon-Weaver diversity index [28]. The data were analyzed by analysis of variance (ANOVA) and Duncan’s new multiple range test (*p* < 0.05) by using SPSS 20.0. Pearson’s correlation test was used to determine the correlation among variables. Graphical representation of the data was performed using GraphPad Prism 8.0.2.

## 3. Results

### 3.1. Variation and Analysis of Main Agronomic Traits of Mung Bean

**Figure 1:**
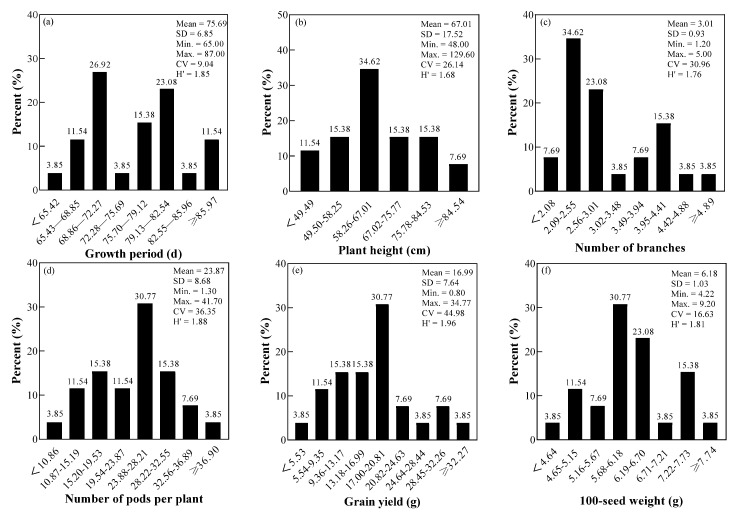
Statistical analysis of six agronomic traits diversity of mung bean cultivars. Note: (**a**) growth period (d); (**b**) plant height (cm); (**c**) number of branches; (**d**) number of pods per plant; (**e**) grain yield (g); (**f**) 100-seed weight (g). SD: standard deviation; CV: coefficient of variation; H’: genetic diversity index.

The results showed that the variation degree of different agronomic traits was different. The variation coefficients of six agronomic traits ranged from 9.04% to 44.98%, and the order was as follows: grain yield > number of pods per plant > number of branches > plant height > 100-seed weight > growth period. This indicated that the grain yield had great inter-individual differences and high screen ability. The average genetic diversity index of six agronomic traits was 1.83, and varied from 1.68 to 1.96. The diversity index of grain yield was the highest, showing rich genetic diversity; however, the diversity index of plant height was the lowest (Figure 1).

It was found that the growth period of 26 mung bean cultivars was mainly distributed in the range of 68.86−72.27 d (26.92%), and the cultivars with growth period less than 65.42 d were less (3.85%). The plant height, number of branches, and number of pods per plant were mostly distributed in the range of 58.26−67.01 cm (34.62%), 2.09−2.55 (34.62%), 23.88−28.21 (30.77%), respectively. The 100-seed weight of mung beans was mainly medium and large. Meanwhile, the grain yield of mung beans was concentrated in the range 17.00−20.81 g (30.77%). The top 5 cultivars with grain yield were: CES-78 (34.8 g), Kelv 2 (29.8 g), (VC3890A/V2709-32-45)-2 (29.4 g), Zhonglv 16 (24.9 g), Bailv 12 (23.1 g).

### 3.2. The Pod Traits of Fresh Beans

**Figure 2:**
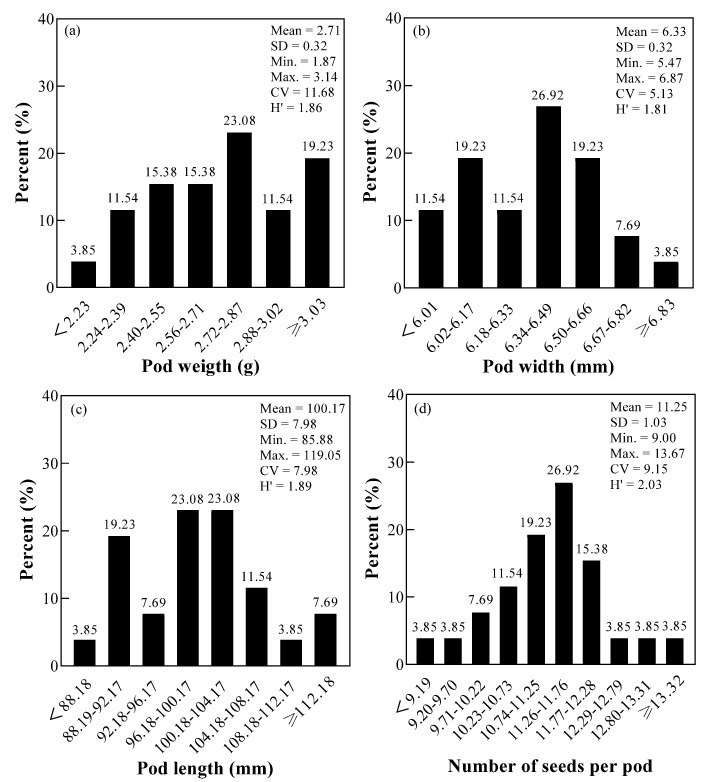
Statistical analysis of pod traits diversity of mung bean cultivars. Note: (**a**) pod weight (g); (**b**) pod width (mm); (**c**) pod length (mm); (**d**) number of seeds per pod.

Figure 2 shows that the variation coefficient of pod traits of fresh mung beans ranged from 5.13% to 11.68%, and the order is: pod weight > number of seeds per pod > pod length > pod width; the diversity index ranged from 1.81 to 2.03, with an average of 1.90, among which the diversity index of number of seeds per pod was the highest, followed by the pod length (1.89). The pod weight, pod width, pod length, and number of pods per plant of 26 mung bean cultivars were mainly distributed in the range of 2.72−2.87 g (23.08%), 6.34−6.49 mm (26.92%), 96.18−104.17 mm (46.15%), 11.26−11.76 (26.92%), respectively. The varieties with the largest pod weight, width, length, and number of seeds per pod were Zhonglv 13-1-1, C01555, Zhanglv 3, C2656, respectively. The cultivars’ higher-than-average pod weight, pod width, pod length, and number of seeds per pod were 14, 16, 12, and 14, respectively.

### 3.3. The Fresh and Boiled Bean Hardness

**Figure 3:**
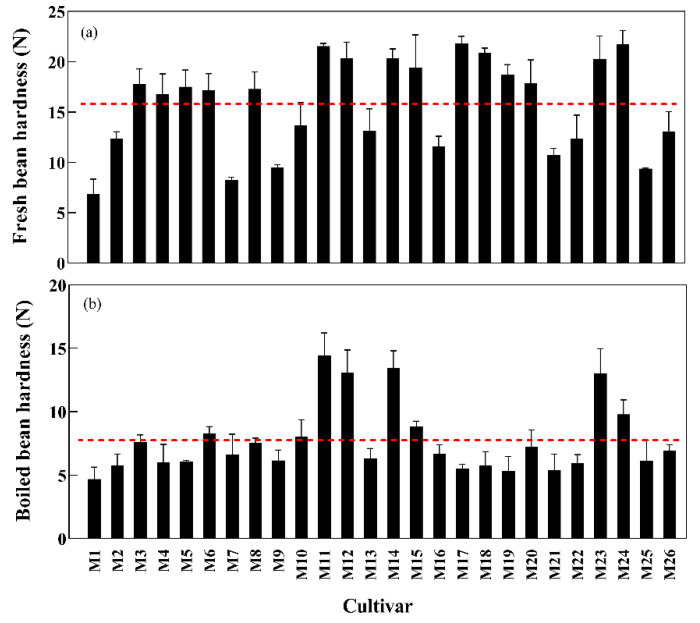
Fresh beans hardness (**a**) and boiled beans hardness (**b**).

Figure 3a shows that the fresh bean hardness of mung bean cultivars ranged from 6.84 to 21.82 N. The hardest and softest cultivars were JL201215 (M17) and Zhonglv 3 (M1), respectively. Fifteen cultivars had a higher-than-average hardness of fresh beans (15.48 N). The hardness of boiled beans in this same sample ranged from 4.69 to 14.42 N, and the cultivars with the hardest and softest hardness were Zhonglv 13-1-1 (M11) and Zhonglv 3 (M1), respectively. Eighteen cultivars had a less-than-average boiled bean hardness (7.72 N) (Figure 3b). The hardness of fresh bean and boiled bean was significantly different among 26 mung bean cultivars (*p* < 0.05) (Table S3).

### 3.4. Sensory Evaluation of Boiled Beans

**Figure 4:**
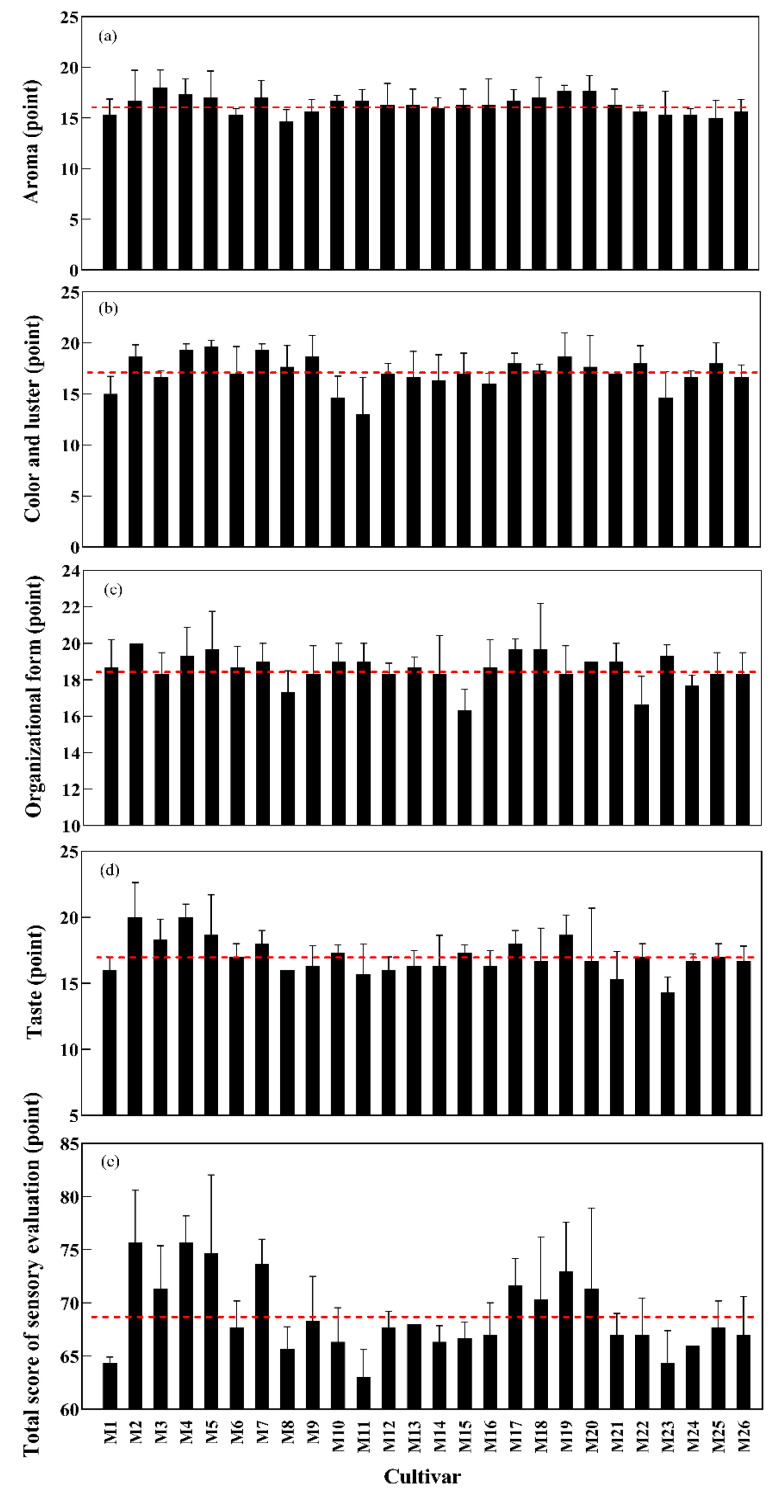
Sensory evaluation of boiled mung beans. Note: (**a**) aroma (point); (**b**) color and luster (point); (**c**) organizational form (point); (**d**) taste (point); (**e**) total score of sensory evaluation (point).

The results showed that the aroma of 26 mung bean cultivars ranged from 14.78 to 18.00 points, and the cultivars with the highest and lowest aroma were Kelv 2 (M3) and VC3890A (M25), respectively (Figure 4a). The color and luster of mung bean cultivars ranged from 12.75 to 19.39 points. The cultivars with the best and worst color and luster were Zhonglv 1 (M5) and Zhonglv 13-1-1 (M11), respectively (Figure 4b). The organizational form in the same sample ranged from 16.33 to 19.89 points, and the cultivars with the best and worst organizational form were Jilv 11 (M2) and Weilv 5 (M15), respectively (Figure 4c). The taste of mung bean cultivars ranged from 14.56 to 20.11 points; the cultivars with the best and worst taste were Zhanglv 3 (M4) and Bailv 12 (M23), respectively (Figure 4d). In general, the total score for sensory evaluation of mung bean cultivars ranged from 63.08 to 75.67 points, nine cultivars had a higher-than-average comprehensive score (68.64 points), and the top five cultivars were Zhanglv 3 (75.67), Jilv 11 (75.39), Zhonglv 1 (74.50), Berkenx109897 (73.47) and Zhonglv 16 (72.92) (Figure 4e). There were no significant differences in aroma and organizational form among 26 mung bean cultivars (*p* > 0.05), but there were significant differences in color and luster, taste and total score for sensory evaluation (*p* < 0.05) (Table S4).

### 3.5. Multivariate Correlation Analysis

**Figure 5:**
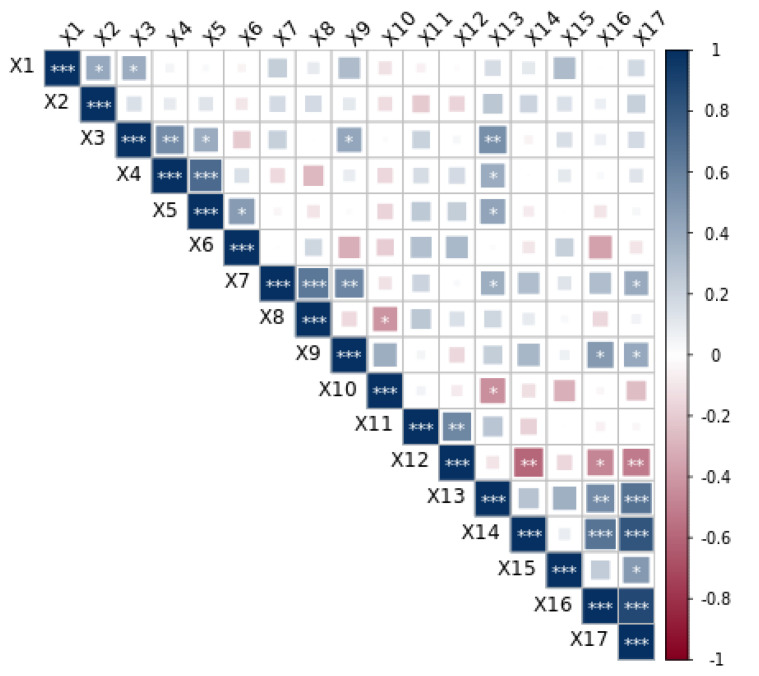
Multivariate correlation of mung bean cultivars. Note: X1: growth period (d); X2: plant height (cm); X3: number of branches; X4: number of pods per plant; X5: grain yield (g); X6: 100-seed weight; X7: pod weight (g); X8: pod width (mm); X9: pod length (mm); X10: number of seeds per pod; X11: fresh bean hardness (N); X12: boiled bean hardness (N); X13: aroma (point); X14: color and luster (point); X15: organizational form (point); X16: taste (point); X17: total score of sensory evaluation (point). *, ** and *** indicate significant difference at *p* < 0.05, 0.01 or 0.001, respectively. Blue represents positive correlation between variables, red represents negative correlation, and the larger the color block, the stronger the correlation.

Correlation results of agronomic traits, fresh food processing characteristics and sensory quality are provided in Figure 5. Results showed that there was a positive correlation between the agronomic traits of mung beans in the maturity stage. Among them, the growth period was related to plant height (r = 0.411, *p* < 0.05) and number of branches (r = 0.396, *p* < 0.05); the number of branches had a significant positive correlation with the number of pods per plant (r = 0.559, *p* < 0.01). In addition, the grain yield was positively correlated with the number of branches (r = 0.399, *p* < 0.05), number of pods per plant (r = 0.717, *P* < 0.01), and 100-seed weight (r = 0.471, *p* < 0.05).

It was found that the pod weight was significantly positively correlated with pod width (r = 0.648, *p* < 0.01), and pod length (r = 0.580, *p* < 0.01). A significant negative correlation was reported between pod width and the number of seeds per pod (r = −0.412, *p* < 0.05). In addition, the fresh bean hardness was significantly correlated with boiled bean hardness (r = 0.572, *p* < 0.01). Significantly, correlation coefficients were reported among boiled bean hardness and sensory evaluation indexes. The results showed that the boiled beans hardness had a significant negative correlation with color and luster (r = −0.593, *p* < 0.01), taste (r = −0.479, *p* < 0.05), and the total score for sensory evaluation (r = −0.513, *p* < 0.01).

### 3.6. Comprehensive Evaluation of Mung Bean Cultivars by Grey Relational Analysis

In the light of market demand and production practice, the reference cultivars were established (Table S5). The higher the correlation with reference cultivars, the better the comprehensive performance of cultivars. The heat map of the influence of various factors on the final result in the grey theory system explained the differences among mung bean cultivars and more intuitively reflected the contribution of each index to the final result, which is helpful in explaining the difference between the weighted correlation degree of the sample (Figure S1). According to the weighted grey relational grade heat map of mung bean cultivars and the weighted correlation degree between 26 cultivars and the reference cultivars, the high-quality cultivars such as Zhanglv 3 (0.960), Jilv 11 (0.942), Zhonglv 1 (0.915), CES-78 (0.899) and Kelv 2 (0.896) were suitable for fresh food processing (Table 2).

## 4. Discussion

The agronomic traits of 26 mung bean cultivars at maturity were analyzed and identified. The coefficient of variation of the five agronomic traits was more than 10%, which indicated that these traits had significant individual differences among different cultivars. Six quantitative characters had rich genetic variation, which was consistent with Qiao et al. [29]. The quantitative characters are controlled by minor genes [30], and the abundant genetic variation of quantitative traits in mung beans indicates that the micro genes are quite different, resulting in a rich genetic diversity index. On the basis of the consistent environmental conditions, the differences among mung bean cultivars are due to their inherent genetic characteristics. The highest coefficient of variation and genetic diversity index was the grain yield, which can be used as the first choice for breeding excellent cultivars; the genetic diversity index of the growth period was higher, but the coefficient of variation was lower, which indicated that the coefficient of variation and the genetic diversity index were not uniform, and other researchers had the same findings on the agronomic traits of mung bean [21]. The length of growth period plays an important role in the morphogenesis of mung bean. The correlation analysis showed that the growth period was positively correlated with the plant height and the number of branches, which was consistent with others’ results. Kamil found that the plant height, number of pods per plant, number of seeds per pod and seed yield per plant of Polish mung bean is 40.45 cm, 9.3, 6.3 and 2.58 g, respectively [8]. The average value of agronomic traits of mung bean cultivars in our study is higher than that of Polish mung bean. The top five cultivars with higher grain yield were CES-78, Kelv 2, (VC3890A/V2709-32-45)-2, Zhonglv 16, Bailv 12, which had more obvious advantages in the number of branches, number of pods per plant, and 100-seed weight. The top five cultivars with higher grain yield were CES-78, Kelv 2, (VC3890A/V2709-32-45)-2, Zhonglv 16, Bailv 12, which had more obvious advantages in the number of branches, number of pods per plant, and 100-seed weight.

Pod weight, pod width, pod length, number of seeds per pod, and fresh bean hardness are the main indexes for breeding fresh mung beans. Different mung bean cultivars had differences in pod traits and fresh bean hardness, which was caused by climatic factors, cultivation and management conditions, growth environment, and genetic characteristics of cultivars [31]. It was found that there was a significant positive correlation between pod weight and pod width of fresh broad bean [32], which was consistent with the results of this experiment: the heavier the fresh pod weight of mung bean, the longer the pod width and length. In addition, the shorter the pod width, the greater the number of seeds per pod.

In order to improve the bioavailability of mung bean nutrients, mung beans are usually processed through different treatments, such as cooking, boiling, baking, roasting, germination, fermentation, and so on [33]. In this study, the fresh mung bean was treated by the common boiling processing method, and the results showed that the hardness of the boiled mung bean was significantly lower than that of untreated fresh mung bean, which may be due to a series of physical and chemical changes in the process of cooking, including gelatinization and swelling of starch, denaturation of protein, solubilization of some polysaccharides, softening of structure, and so on [22]. Several studies reported that boiled mung bean was more conducive to increasing soluble fiber content, lowering cholesterol, enhancing antioxidant activity, and inhibiting weight gain and fat accumulation, more suitable as a food supplement for patients with hypercholesterolemia [34,35]. Because of the lower texture parameters and higher nutritional health benefits of boiled mung bean, it is more likely to appeal to middle-aged and elderly consumers [23,36].

Aroma, color and luster, organizational form and taste are all vital factors for sensory evaluation. Results showed that the boiled bean hardness had the strongest correlation with color, luster and taste. The softer the mung bean hardness with bright color and soft taste, the higher the sensory evaluation score. Taste is a considerable attribute to describe food quality. In this study, the taste of boiled beans was significantly positively correlated with aroma and color, and had the highest correlation with the total score of sensory evaluation (r = 0.802), which intuitively reflected the evaluators’ acceptance and preference for boiled beans. Mung bean cultivars with excellent sensory quality are more suitable for promotion to the market.

The cultivars suitable for fresh food processing were screened out more quickly and conveniently through grey relational analysis: Zhanglv 3, Jilv 11, Zhonglv 1, CES-78, Kelv 2, which was basically consistent with the results of sensory evaluation. It is worth noting that Zhanglv 3, Jilv 11, Zhonglv 1, Berkenx109897, JL201215, and BL13-637 cultivars were suitable for fresh food processing, although their yield was not high. Previous studies mostly used grey relational analysis to evaluate the agronomic traits or nutritional quality of crops [26]. In this study, combined with the yield and fresh food processing characteristics, high-quality cultivars CES-78, Kelv 2, Zhonglv 16, and Zhonglv 2 were selected by comprehensive evaluation. In the future, the nutritional quality and functional characteristics of ideal cultivars can be studied in order to explore their potential application value. Other mung bean cultivars can improve their palatability, sensory quality and commerciality through different processing methods and conditions.

## 5. Conclusions

By analyzing the yield and fresh processing characteristics of 26 mung bean cultivars, CES-78, Kelv 2, (VC3890A/V2709-32-45)-2, Zhonglv 16, Bailv 12 cultivars were screened out with higher yield; Zhanglv 3, Jilv 11, Zhonglv 1, CES-78, Kelv 2 were suitable for fresh processing. According to the comprehensive evaluation, the cultivars with high yield and excellent fresh food processing characteristics were CES-78, Kelv 2, Zhonglv 16, and Zhonglv 2. This study provided a scientific basis for the breeding of mung bean cultivars, development of new products, and improvement of mung bean resource utilization. In the future, the nutritional quality and functional characteristics of high-quality cultivars can be studied to explore their potential application value.

## Figures and Tables

**Table 1:** Names of 26 mung bean cultivars.

Code	Cultivar	Code	Variety
M1	Zhonglv 3	M14	Sulv 5
M2	Jilv 11	M15	Weilv 5
M3	Kelv 2	M16	Weilv 6
M4	Zhanglv 3	M17	JL201215
M5	Zhonglv 1	M18	BL13-637
M6	Zhonglv 2	M19	Zhonglv 16
M7	Berkenx109897	M20	CES-78
M8	VC1560A	M21	Lvfeng 3
M9	Lvfeng 5	M22	C2656
M10	Elv 1	M23	Bailv 12
M11	Zhonglv 13-1-1	M24	VC1482A
M12	(VC3890A/V2709-32-45)-2	M25	VC3890A
M13	(VC3890A/TC1966)-3-2001-541	M26	C01555

**Table 2:** Weighted grey relational degree of 26 mung bean cultivars.

Sort	Cultivar	WGRD	Sort	Cultivar	WGRD
1	Zhanglv 3	0.960	14	Elv 1	0.861
2	Jilv 11	0.942	15	Weilv 5	0.856
3	Zhonglv 1	0.915	16	VC3890A	0.855
4	CES-78	0.899	17	VC1560A	0.851
5	Kelv 2	0.896	18	(VC3890A/TC1966)-3-2001-541	0.851
6	Zhonglv 16	0.895	19	Lvfeng 3	0.846
7	Zhonglv 2	0.879	20	Weilv 6	0.843
8	BL13-637	0.875	21	VC1482A	0.837
9	Berkenx109897	0.874	22	(VC3890A/V2709-32-45)-2	0.832
10	JL201215	0.872	23	Sulv 5	0.825
11	C2656	0.868	24	Zhonglv 13-1-1	0.812
12	C01555	0.867	25	Bailv 12	0.812
13	Lvfeng 5	0.864	26	Zhonglv 3	0.805

WGRD: Weighted grey relational degree.

## Data Availability

The data presented in this study are available on request from the corresponding author.

## Supplementary Materials

The following supporting information can be downloaded at: https://www.mdpi.com/article/10.3390/foods11121687/s1, Figure S1: The weighted grey relational grades (WGRG) heat map of mung bean varieties-fresh food processing; Table S1: The emergence, florescence, maturity data of mung beans; Table S2: Sensory evaluation standard of boiled beans; Table S3: The fresh and boiled bean hardness; Table S4: Sensory evaluation of boiled mung beans; Table S5: The characteristics of all mung beans and the perfect sample.
