# Supplementary material for: Agronomic Traits, Fresh Food Processing Characteristics and Sensory Quality of 26 Mung Bean (Vigna radiata L.) Cultivars (Fabaceae) in China
Source: Foods. 2022 Jun 8;11(12):1687. doi: 10.3390/foods11121687 (PMC9222593; doi:10.3390/foods11121687)
Supplement: Supplementary file 1 [file foods-11-01687-s001.zip › foods-1721036-supplementary.pdf]

Table S1. The emergence, florescence, maturity data of mung beans.

Code	Variety	Emergence	Florescence	Maturity
M1	Zhonglv 3	6.26	7.3	9.3
M2	Jilv 11	6.25	8.1	9.9
M3	Kelv 2	6.25	7.28	9.3
M4	Zhanglv 3	6.26	8.9	9.21
M5	Zhonglv 1	6.26	7.29	9.5
M6	Zhonglv 2	6.26	7.3	9.4
M7	Berkenx109897	6.26	8.2	9.1
M8	VC1560A	6.26	8.4	9.14
M9	Lvfeng 5	6.26	8.5	9.16
M10	Elv 1	6.26	8.1	9.14
M11	Zhonglv 13-1-1	6.26	7.26	8.3
M12	(VC3890A/V2709-32-45)-2	6.26	8.6	9.21
M13	(VC3890A/TC1966)-3-2001-541	6.26	8.6	9.15
M14	Sulv 5	6.26	8.6	9.14
M15	Weilv 5	6.26	7.26	9.3
M16	Weilv 6	6.26	7.25	9.2
M17	JL201215	6.26	7.27	9.3
M18	BL13-637	6.26	7.25	9.2
M19	Zhonglv 16	6.26	7.27	9.8
M20	CES-78	6.26	8.3	9.13
M21	Lvfeng 3	6.26	7.25	9.2
M22	C2656	6.26	8.2	9.17
M23	Bailv 12	6.26	7.27	9.3
M24	VC1482A	6.26	8.4	9.15
M25	VC3890A	6.26	8.3	9.13
M26	C1555	6.26	8.3	9.21

Table S2. Sensory evaluation standard of boiled bean.

Score / Index	25~19	18~13	12~7	6~0
Color and lustre	Glamorous	Slightly glamorous	Dim	Dimmer
Aroma	Full-bodied fragrance of beans	With the fragrance of beans	No aroma	Rotten smell
Tissue morphology	The surface is smooth and wrinkle-free	Wrinkle slightly	Wrinkle	The tissue is seriously damaged
Taste	Soft	Slightly soft	Slightly hard	Hard

Table S3. The fresh beans hardness and boiled beans hardness.

Variety	Fresh bean hardness (N)	Bolied bean hardness (N)
M1	6.84±1.50k	4.69±0.94h
M2	12.33±0.71fgh	5.77±0.90fgh
M3	17.81±1.47cde	7.61±0.56cdef
M4	16.81±2.00e	6.00±1.43efgh
M5	17.51±1.67cde	6.07±0.10efgh
M6	17.15±1.66e	8.31±0.51bcd
M7	8.27±0.27jk	6.61±1.62defgh
M8	17.29±1.69de	7.57±0.36cdefg
M9	9.51±0.27hijk	6.16±0.82defgh
M10	13.71±2.27f	8.03±1.32bcde
M11	21.55±0.26ab	14.42±1.80a
M12	20.33±1.63abc	13.07±1.79a
M13	13.13±2.20fg	6.33±0.77defgh
M14	20.38±0.89abc	13.45±1.36a
M15	19.44±3.22abcde	8.81±0.45bc
M16	11.63±0.97fghi	6.67±0.72cdefgh
M17	21.82±0.71a	5.49±0.38fgh
M18	20.87±0.49ab	5.79±1.07fgh
M19	18.74±0.97bcde	5.32±1.16gh
M20	17.88±2.29cde	7.25±1.34cdefg
M21	10.70±0.69ghij	5.37±1.27fgh
M22	12.39±2.30fgh	5.97±0.65efgh
M23	20.27±2.30abcd	13.03±1.94a
M24	21.74±1.37ab	9.79±1.15b
M25	9.39±0.04ijk	6.12±1.65defgh
M26	13.10±1.95fg	6.93±0.45cdefg

Table S4. Sensory evaluation of boiled mung beans.

Variety	Aroma	Color and luster	Organizational form	Flavor	Total score of sensory evaluation
M1	15.33±1.53ab	15.00±1.73bcd	18.67±1.53abcd	16.00±1.00bc	64.33±0.58fg
M2	16.67±3.06ab	18.67±1.15ab	20.00±0.00a	20.00±2.65a	75.67±4.93a
M3	18.00±1.73a	16.67±0.58abc	18.33±1.15abcd	18.33±1.53ab	71.33±4.04abcdef
M4	17.33±1.53ab	19.33±0.58a	19.33±1.53ab	20.00±1.00a	75.67±2.52a
M5	17.00±2.65ab	19.67±0.58a	19.67±2.08ab	18.67±3.06ab	74.67±7.37ab
M6	15.33±0.58ab	17.00±2.65abc	18.67±1.15abcd	17.00±1.00abc	67.67±2.52cdefg
M7	17.00±1.73ab	19.33±0.58a	19.00±1.00abc	18.00±1.00ab	73.67±2.31abc
M8	14.67±1.15b	17.67±2.08abc	17.33±1.15bcd	16.00±0.00bc	65.67±2.08efg
M9	15.67±1.15ab	18.67±2.08ab	18.33±1.53abcd	16.33±1.53bc	68.33±4.16bcdefg

M10	16.67±0.58ab	14.67±2.08cd	19.00±1.00abc	17.33±0.58abc	66.33±3.21defg
M11	16.67±1.15ab	13.00±3.61d	19.00±1.00abc	15.67±2.31bc	63.00±2.65g
M12	16.33±2.08ab	17.00±1.00abc	18.33±0.58abcd	16.00±1.00bc	67.67±1.53cdefg
M13	16.33±1.53ab	16.67±2.52abc	18.67±0.58abcd	16.33±1.15bc	68.00±0.00bcdefg
M14	16.00±1.00ab	16.33±2.52abcd	18.33±2.08abcd	16.33±2.31bc	66.33±1.53defg
M15	16.33±1.53ab	17.00±2.00abc	16.33±1.15d	17.33±0.58abc	66.67±1.53cdefg
M16	16.33±2.52ab	16.00±1.00abcd	18.67±1.53abcd	16.33±1.15bc	67.00±3.00cdefg
M17	16.67±1.15ab	18.00±1.00abc	19.67±0.58ab	18.00±1.00ab	71.67±2.52abcde
M18	17.00±2.00ab	17.33±0.58abc	19.67±2.52ab	16.67±2.52abc	70.33±5.86abcdef
M19	17.67±0.58ab	18.67±2.31ab	18.33±1.53abcd	18.67±1.53ab	73.00±4.58abcd
M20	17.67±1.53ab	17.67±3.06abc	19.00±0.00abc	16.67±4.04abc	71.33±7.57abcdef
M21	16.33±1.53ab	17.00±0.00abc	19.00±1.00abc	15.33±2.08bc	67.00±2.00cdefg
M22	15.67±0.58ab	18.00±1.73abc	16.67±1.53cd	17.00±1.00abc	67.00±3.46cdefg
M23	15.33±2.31ab	14.67±2.52cd	19.33±0.58ab	14.33±1.15c	64.33±3.06fg
M24	15.33±0.58ab	16.67±0.58abc	17.67±0.58abcd	16.67±0.58abc	66.00±0.00defg
M25	15.00±1.73ab	18.00±2.00abc	18.33±1.15abcd	17.00±1.00abc	67.67±2.52cdefg
M26	15.67±1.15ab	16.67±1.15abc	18.33±1.15abcd	16.67±1.15abc	67.00±3.61cdefg

Table S5. The characteristics of all mung beans and the perfect sample.

	Ideal Vari-ety	M1	M2	M3	M4	M5	M6	M7	M8	M9	M10	M11	M12	M13	M14	M15	M16	M17	M18	M19	M20	M21	M22	M23	M24	M25	M26
X1	3.30	1.87	3.09	2.92	2.89	3.13	2.65	2.54	2.31	2.82	2.26	3.14	2.61	2.36	2.51	2.84	2.69	2.41	2.82	2.86	3.10	2.74	2.56	2.43	2.74	2.98	3.12
X2	7.21	5.47	6.17	6.40	5.79	6.68	6.13	6.45	6.21	6.09	5.79	6.77	6.48	6.38	6.09	6.52	6.48	6.50	6.37	6.49	6.60	6.62	6.02	6.28	6.33	6.63	6.87
X3	125.00	88.94	117.75	104.38	119.05	99.33	103.10	85.88	94.41	109.92	99.29	100.27	95.23	91.47	102.19	98.97	91.91	92.10	103.36	99.32	106.51	103.02	106.78	90.01	99.83	100.12	101.27
X4	14.35	12.33	11.11	10.44	12.11	9.89	12.89	9.00	10.89	11.44	11.67	11.22	10.44	9.89	11.00	11.44	9.44	11.67	11.56	11.33	10.67	11.11	13.67	11.33	12.11	12.00	11.78
X5	22.91	6.84	12.33	17.81	16.81	17.51	17.15	8.27	17.29	9.51	13.71	21.55	20.33	13.13	20.38	19.44	11.63	21.82	20.87	18.74	17.88	10.70	12.39	20.26	21.74	9.39	13.10
X6	4.46	4.69	5.77	7.61	6.00	6.07	8.31	6.61	7.56	6.16	8.03	14.42	13.07	6.33	13.45	8.81	6.67	5.49	5.79	5.32	7.25	5.37	5.97	13.03	9.79	6.12	6.93
X7	18.90	15.06	16.78	18.00	17.33	16.72	15.67	17.19	14.86	15.44	16.39	16.42	16.53	16.22	15.72	16.44	16.33	16.50	16.83	17.44	17.72	16.11	15.56	15.56	15.44	14.78	15.39
X8	20.36	14.72	18.78	16.78	19.06	19.39	16.67	19.25	17.64	18.56	14.50	12.75	16.81	16.56	16.11	16.97	15.89	17.94	17.44	18.58	17.78	16.75	18.00	14.67	16.31	18.00	16.69
X9	20.88	18.56	19.89	17.94	19.17	19.78	18.44	18.83	17.00	18.11	18.92	18.67	18.36	18.67	18.06	16.33	18.56	19.64	19.56	18.22	18.83	18.64	16.56	19.11	17.33	18.11	18.14
X10	21.12	15.94	19.94	18.33	20.11	18.61	16.89	18.19	16.00	16.44	17.44	15.25	15.92	16.44	16.17	17.50	16.22	17.78	16.67	18.67	16.61	15.14	17.11	14.56	16.47	16.89	16.50
X11	79.45	64.28	75.39	71.06	75.67	74.50	67.67	73.47	65.50	68.56	66.17	63.08	67.61	67.89	66.06	66.75	67.00	71.86	70.25	72.92	70.94	66.64	67.22	64.22	65.56	67.78	66.72

	X7	X8	X9	X10	X11	X12	X13	X14	X15	X16	X17
M1	0.333	0.333	0.355	0.636	0.409	0.542	0.354	0.416	0.571	0.400	0.356
M2	0.934	0.499	0.927	0.477	0.692	0.652	0.569	0.845	1.000	0.943	0.958
M3	0.740	0.598	0.531	0.420	0.845	1.000	1.000	0.560	0.478	0.610	0.577
M4	0.719	0.392	1.000	0.600	1.000	0.682	0.707	0.909	0.711	1.000	1.000
M5	0.986	0.785	0.457	0.382	0.886	0.691	0.558	1.000	0.941	0.649	0.844
M6	0.563	0.485	0.510	0.750	0.941	0.876	0.408	0.549	0.552	0.463	0.440
M7	0.514	0.622	0.333	0.333	0.458	0.776	0.667	0.960	0.627	0.592	0.741
M8	0.433	0.514	0.402	0.457	0.919	0.986	0.339	0.655	0.381	0.403	0.382
M9	0.662	0.471	0.645	0.512	0.510	0.704	0.387	0.799	0.500	0.431	0.469
M10	0.417	0.392	0.456	0.538	0.837	0.942	0.500	0.404	0.646	0.510	0.398
M11	1.000	0.876	0.469	0.488	0.536	0.344	0.504	0.333	0.593	0.364	0.333
M12	0.544	0.639	0.410	0.420	0.609	0.397	0.523	0.562	0.538	0.398	0.439
M13	0.448	0.586	0.376	0.382	0.769	0.730	0.475	0.540	0.593	0.431	0.447
M14	0.502	0.472	0.496	0.467	0.606	0.381	0.414	0.503	0.492	0.413	0.396
M15	0.682	0.665	0.452	0.512	0.675	0.777	0.509	0.579	0.333	0.515	0.414
M16	0.586	0.639	0.379	0.356	0.635	0.786	0.492	0.487	0.571	0.417	0.421
M17	0.464	0.653	0.381	0.538	0.522	0.620	0.518	0.697	0.877	0.543	0.623
M18	0.662	0.584	0.514	0.525	0.574	0.655	0.580	0.631	0.842	0.446	0.537
M19	0.692	0.649	0.457	0.500	0.739	0.601	0.744	0.805	0.516	0.658	0.696
M20	0.948	0.722	0.569	0.438	0.837	0.905	0.853	0.673	0.627	0.442	0.571
M21	0.615	0.732	0.509	0.477	0.574	0.607	0.460	0.557	0.587	0.358	0.411
M22	0.520	0.451	0.575	1.000	0.697	0.678	0.397	0.705	0.348	0.481	0.427
M23	0.472	0.541	0.363	0.500	0.613	0.399	0.397	0.413	0.696	0.333	0.355
M24	0.615	0.566	0.463	0.600	0.526	0.637	0.387	0.518	0.410	0.433	0.384
M25	0.798	0.742	0.467	0.583	0.505	0.698	0.333	0.705	0.500	0.463	0.444
M26	0.967	1.000	0.483	0.553	0.766	0.836	0.382	0.552	0.504	0.435	0.413

Figure S1. The weighted grey relational grades (WGRG) heat map of mung bean varieties-fresh food processing.
